# Emerging innovations in theranostics for pancreatic neuroendocrine tumors

**DOI:** 10.1038/s41698-025-00938-1

**Published:** 2025-05-19

**Authors:** Anita Karimi, Christina Bogdani, Elisabeth O’Dwyer, Despina Siolas

**Affiliations:** 1 Department of Medicine, Division of Hematology and Oncology, Weill Cornell Medicine, New York, NY USA; 2 New York Institute of Technology College of Osteopathic Medicine, Old Westbury, New York, NY USA; 3 Division of Molecular Imaging and Therapeutics, Department of Radiology, Weill Cornell Medicine, New York, NY USA; 4 Sandra and Edward Meyer Cancer Center, Weill Cornell Medicine, New York, NY USA

**Keywords:** Cancer imaging, Radiotherapy, Pancreatic cancer, Targeted therapies

## Abstract

Pancreatic neuroendocrine tumors (pNETs) often overexpress somatostatin receptor type 2 (SSTR2), making them ideal targets for theranostics, which integrates molecular imaging with targeted radionuclide therapy. ^177^Lu-DOTATATE significantly extends progression-free survival (22.8 vs. 8.5 months) compared to octreotide LAR. Despite these advances, challenges remain, including treatment resistance and long-term toxicities. In this review, we explore advancements in specialized imaging techniques, rationale combination strategies, and exploring next-generation radiopharmaceuticals.

## Introduction

Pancreatic neuroendocrine tumors (pNETs) are the second most common type of neuroendocrine tumor (NET)^1,2^, accounting for approximately 8-10% of all pancreatic neoplasms^3^. The incidence of pNETs has more than doubled over the past three decades, largely due to advances in radiographic detection, although the contributions of genetic and environmental factors are still under investigation^1,4^. Despite diagnostic advancements, managing pNETs remains challenging due to their heterogeneous behavior, and limited systemic treatment options. Systemic therapy for pNETs has traditionally focused on somatostatin analogs (SSA) due to the high expression of somatostatin receptor type 2 (SSTR2) on tumor cells. While SSAs are effective at controlling hormonal symptoms and stabilizing disease, they rarely induce significant tumor regression, highlighting a critical unmet clinical need for therapies that actively reduce tumor burden. The development of peptide receptor radionuclide therapy (PRRT) has introduced a promising therapeutic strategy by leveraging SSTR2 expression to enable highly sensitive imaging of small tumors and the delivery of targeted radiation directly to tumor cells. This review explores recent advances in specialized imaging techniques, PRRT-based combination therapies, and emerging radiopharmaceutical agents for the treatment of pNETs.

## Biology of pNETs and the role of SSTR

pNETs originate from pancreatic islet cells and exhibit diverse biological behaviors, from indolent to highly aggressive. Approximately 75–90% of pNETs are non-functional, meaning they do not secrete metabolically active hormones, leading to asymptomatic progression and delaying diagnosis until after metastasis has occurred^1,5^. In contrast, functional tumors can secrete hormones, such as glucagon, insulin, and gastrin, causing distinct clinical syndromes that can prompt earlier detection^6^. Somatostatin, a hormone produced by neuroendocrine cells, regulates glucagon and insulin secretion and influences pancreatic cell growth through multiple signaling pathways^7,8^. A key biological characteristic of pNETs is the overexpression of somatostatin receptors (SSTRs), particularly SSTR2, which is found in around 80% of non-functional pNETS^9^. The high expression of SSTR2 in well-differentiated pNETs, while poorly differentiated tumors tend to exhibit lower levels of this receptor^10^. This differential expression of SSTR2 not only serves as a diagnostic marker but also presents a valuable therapeutic target. High SSTR2 expression is associated with a favorable response to SSAs due to their preferential binding to SSTR2^11^. Consequently, SSAs are widely employed to control tumor growth and alleviate hormonal symptoms in patients^12^. The limited efficacy of SSAs in causing significant tumor regression combined treatment resistance, underscores the necessity for more targeted and effective treatment strategies. The availability of a tumor-specific target (SSTR2) combined with advances in nuclear medicine has paved the way for theranostics, an approach that integrates molecular imaging and targeted radionuclide therapy to offer a more precise and effective treatment strategy.

## Specialized imaging

While traditional imaging modalities such as computed tomography (CT) and magnetic resonance imaging (MRI) are commonly used for pNET detection, their sensitivity is often limited, especially for small or well-differentiated lesions. In contrast, molecular imaging techniques utilizing radiolabeled somatostatin analogs have significantly enhanced the ability to localize and assess tumor activity with higher precision. The Octreoscan (Mallinckrodt, St. Louis, MO), the first Food and Drug Administration (FDA)-approved imaging modality for pNETs, utilizes planar and SPECT/CT imaging performed 24 and 36 h after the intravenous administration of ^111^In-pentetreotide, a gamma-emitting radiotracer targeting SSTR2. However, since the FDA approval of ^68^Ga-DOTATATE Positron Emission Tomography-Computed Tomography (PET/CT) in June 2016, this tracer has largely replaced Octreoscan due to its superior sensitivity (96–97%) which is significantly higher than ^111^In-pentetreotide SPECT/CT (65–72%), and comparable specificity (93%) in detecting SSTR2-expressing tumors^13^. This shift is also driven by reduced radiation exposure and a shorter imaging protocol, as ^68^Ga-DOTATATE scans are completed within 1–2 h^14–20^.

^68^Ga is a positron-emitting isotope chelated to 1,4,7,10-tetraazacyclododecane-1,4,7,10-tetraacetic acid (DOTA), which is conjugated to peptides with high affinity for SSTRs. Common ^68^Ga compounds include DOTATOC, DOTANOC, and DOTATATE^21^. While these tracers show minor differences in binding affinities to specific SSTR subtypes, they exhibit comparable diagnostic accuracy for detecting neuroendocrine tumors (NETs)^22,23^. ^68^Ga-DOTATATE PET/CT is the most widely used in the United States, while DOTATOC and DOTANOC are more commonly utilized in Europe. ^68^Ga-DOTATATE PET/CT is particularly effective for imaging well-differentiated, low-grade pNETs due to their high SSTR2 expression, whereas poorly differentiated, grade 3 pNETs, with reduced SSTR expression and increased glycolytic activity, are better evaluated using ^18^F-fluorodeoxyglucose (^18^F-FDG) PET/CT imaging^23^.

### Emerging radiotracers

Among newer agents, ^64^Cu-DOTATATE, approved in the U.S. since 2020, offers significant advantages over ^68^Ga-based tracers, including a longer half-life of 12.7 h, compared to 68 min for ^68^Ga^24–26^.This extended half-life allows for greater flexibility in imaging schedules and facilitates centralized production and distribution to sites without on-site cyclotron facilities^27^. ^64^Cu-DOTATATE also provides lower radiation exposure and improved lesion detection^25^. A comparative study revealed that ^64^Cu-DOTATATE detected 42 additional lesions compared to and ^68^Ga-DOTATOC, 33 of which were confirmed as true positives (*p* < 0.001), demonstrating its superior sensitivity^28^. Despite these benefits, ^64^Cu-DOTATATE has limitations, including higher production costs and longer imaging times (1–3 h) compared to 68Ga-DOTATATE^28^. While these factors may limit widespread adoption, current evidence is inconclusive regarding the superiority of one over another, and both remain valuable diagnostic options in clinical practice.

Current SSTR-mediated imaging relies on the internalization of radiolabeled agonists into tumor cells. However, preclinical studies suggest that radiolabeled SSTR antagonists may offer superior tumor uptake and detection due to enhanced receptor recognition^29,30^. For instance, ^68^Ga-DOTA-JR11, an SSTR antagonist, demonstrated 1.3 times higher tumor uptake compared to ^68^Ga-DOTATATE, despite having 150 times lower affinity for SSTR2^29^. Early human studies using ^68^Ga-DOTA-JR11 have shown favorable safety profiles, optimal biodistribution, and the ability to detect lesions as small as 1.4 mL^17^. SSTR antagonists may provide a more comprehensive assessment of tumor burden in pNET patients.

### PET imaging techniques: dual-tracer PET and hybrid imaging

Dual-tracer PET imaging has emerged as a valuable approach for staging and grading pNETs, particularly those with heterogeneous features. This technique combines the high sensitivity of ^68^Ga-DOTATATE for detecting well-differentiated, SSTR-positive tumors with the ability of ^18^F-FDG PET/CT to identify glycolytically active, poorly differentiated tumors^31^. In a recent study involving 124 patients with Grade 1 and Grade 2 pNETs (49.2%/50.8%, respectively), ^68^Ga-DOTATOC PET/CT detected disease in 122 patients (98.4%), while ^18^F-FDG PET/CT identified lesions in 64 patients (51.6%), resulting in a combined sensitivity of 99.2%^32^. This dual-tracer approach not only enhances diagnostic accuracy but also improves treatment stratification by distinguishing SSTR-positive, indolent tumors from highly glycolytic, aggressive subtypes, allowing for targeted treatment planning^31,33–35^. Similarly, hybrid imaging techniques such as PET/MRI can further refine staging accuracy by integrating the functional insights of PET with the high-resolution anatomical detail of MRI^36^. This combination is particularly effective for detecting small liver metastases and peritoneal implants that might be missed by conventional imaging^36,37^. For example, PET/MRI has shown superior performance in identifying sub-centimeter liver lesions and evaluating vascular invasion, thereby providing more precise information for surgical planning^37^.

Collectively, advanced imaging techniques address the limitations of traditional imaging by offering more precise staging and a deeper understanding of tumor heterogeneity, thereby enabling more personalized treatment approaches.

## Current treatment approaches for pNETS

Surgical resection for localized or limited metastatic disease remains the primary potentially curative option for patients with pNETs. However, the majority of patients (60.2%) present with metastatic disease, while an additional 20.7% are diagnosed with regionally advanced tumors^38^, necessitating a focus on reducing tumor burden, and managing symptoms through multimodal strategies. These strategies often include a combination of surgery, liver-directed treatments, and systemic therapies. The following sections explore key systemic therapies, highlighting their roles in the comprehensive management of pNETs (see Table 1).

**Table 1:** Landmark Clinical Trials for the Treatment of GEP-NETs

Trial Name/ID/Study design	Primary endpoint	Result	Dates of Enrollment	Number Enrolled	Status
PROMID /NCT00171873/Randomized, Double blind Phase III	Time to Tumor Progression	Time to tumor progression: Octreotide cohort- 14.3 mo (CI 95% 11.0 to 28.8) Placebo cohort- 6 mo (CI 95% 3.7 to 9.4)	2001–2013	85	Completed
NCT00353496/ CLARINET/Phase III, Randomised, Double-blind	PFS	Median PFS: Lanreotide cohort- was not reached Placebo cohort- 72 weeks (CI 95% 48.6 to 96.0)	2006–2013	264	Completed
NCT00842348/Phase III Open Label Extension Study of Lanreotide Autogel 120 mg	AE	AE: 86 out of 89 patients with treatment emergent AEs PFS: Lanreotide-Lanreotide cohort: 154.14 weeks (CI 95%, 123.57 to 237.43) Placebo-Lanreotide cohort: 72.00 weeks (CI 95%, 48.43 to 84.57)	2009–2015	89	Completed
NCT01824875/Randomized Phase II	PFS	PFS: Temozolomide cohort- 15.1 months (CI 95% 10.5 to 21.0) Temozolomide and Capecitabine (CAPTEM) cohort- 23.2 months (CI 95% 16.6 to 32.2)	2013–2023	144	Active, not recruiting
NCT01578239/ NETTER-1/Randomized Phase III	PFS	PFS: 177Lu-DOTATATE cohort- at month 20 was 65.2% (95% CI, 50.0 to 76.8) LAR Octreotide cohort- at month 20, 10.8% (95% CI, 3.5 to 23.0) P = 0.004 ORR: 177Lu-DOTATATE- 18% (CI 95% 7.8 to 21.6) LAR Octreotide cohort- 3% (CI 95% 0.2 to 7.8) OS: ^177^Lu-DOTATATE cohort- 48 months (CI 95% 37.4 to 55.2) LAR Octreotide cohort- 36.3 months (CI 95% 25.9 to 51.7) (HR 0.84, 95% CI 0.60-1.17. *p* value 0.3039)	2012–2021	231	Completed

### Somatostatin analogs

SSAs are a widely used first-line treatment for patients with advanced NETs that express SSTRs, due to their anti-proliferative effects and efficacy in symptom management^39^. Common SSAs include octreotide long-acting release (LAR) and lanreotide, both of which have demonstrated significant improvements in progression-free survival (PFS) in separate Phase III clinical trials^40,41^. The CLARINET trial (NCT00353496), which evaluated the efficacy of lanreotide in patients with gastroenteropancreatic NETs (GEP-NETs), reported a median PFS that was not reached in the lanreotide group compared to 18.0 months in the placebo group (HR 0.47; 95% CI, 0.30–0.73; *p* < 0.001)^42^. This trial also included a subgroup analysis focused specifically on pNET patients, which demonstrated a favorable trend for disease control in those receiving lanreotide (HR = 0.58; 95% CI, 0.32–1.04)^43^. While SSAs are effective for disease stabilization, they have limitations, including treatment resistance, incomplete tumor control, and gastrointestinal side effects.

### Peptide receptor radionucleotide therapy (PRRT)

**Fig. 1:**
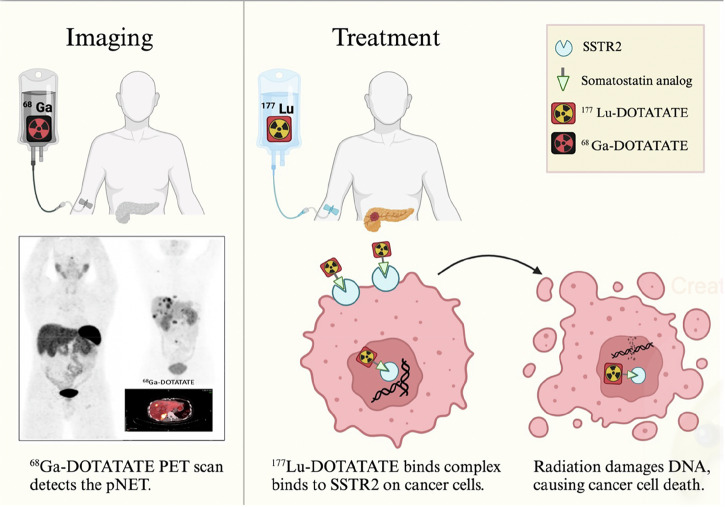
Radionuclide Detection and Treatment of pNETs. This figure illustrates the radionuclide-based approach for detecting and treating pancreatic neuroendocrine tumors (pNETs). The process begins with the intravenous injection of ^68^Ga-DOTATATE, a somatostatin receptor type 2 (SSTR2) agonist that binds to receptors expressed on the surface of neuroendocrine tumor (NET) cells. A subsequent PET/CT scan detects NETs by visualizing this receptor-ligand interaction. For treatment, patients receive ^177^Lu-DOTATATE intravenously as part of peptide receptor radionuclide therapy (PRRT). The ^177^Lu-DOTATATE binds specifically to SSTR2 receptors on neuroendocrine cancer cells, is internalized, and delivers targeted radiation directly to the tumor. This radiation induces DNA damage, leading to cancer cell death while sparing surrounding healthy tissues. Created in Biorender. Karimi, A. (2025).

PRRT (Fig. 1) utilizes radiolabeled somatostatin analogs by linking the β-emitting radioisotope ^177^Lutetium (^177^Lu) to the somatostatin analog octreotate through a DOTA chelator, enabling targeted radiation delivery to tumors with high SSTR expression^44^. The NETTER-1 trial (NCT01578239)was the first phase III trial to assess the efficacy and safety of ^177^Lu-DOTATATE in patients with metastatic or inoperable, well-differentiated SSTR positive midgut NETs that had progressed despite prior SSA therapy^45^. The trial demonstrated that ^177^Lu-DOTATATE reduced the risk of progression or death by 79% compared to high-dose octreotide LAR, with a 20-month progression-free survival (PFS) rate of 65.2% for the ^177^Lu-DOTATATE group versus 10.8% for the control group^45^. In addition, the objective response rate (ORR) was significantly higher in the ^177^Lu-DOTATATE group (18%) compared to 3% in the control group (*P* < 0.001)^45^. Although OS was longer in the PRRT group, the difference between the two groups did not reach statistical significance^46^. Given the favorable PFS, and significant objective response rate observed in patients with midgut neuroendocrine tumors in the NETTER-1 trial, the FDA extended ^177^Lu-DOTATATE approval to encompass all gastrointestinal and pancreatic neuroendocrine tumors^47^. ^177^Lu-DOTATATE is generally well tolerated, but grade 3 or 4 nephrotoxicity has been reported in 2–3% of patients, grade 3 or 4 bone marrow toxicity in 9% as well as Myelodysplastic syndrome (MDS), leukemia at 2% and 3%, respectively^48^. To mitigate nephrotoxicity, co-infusion of positively charged amino acids (e.g., L-lysine and L-arginine) can reduce renal radiation dose by up to 65%^48^. Regular monitoring of renal function and blood counts is essential for early detection of complications^49^.

The follow-up Phase III NETTER-2 trial (NCT03972488) is the first randomized study to assess radioligand therapy as a first-line treatment^50^. Results from the trial indicate that combining ^177^Lu-DOTATATE with octreotide LAR substantially improved PFS (22.8 months, 95% CI: 19.4–not estimated) compared to octreotide LAR alone (8.5 months, 95% CI: 7.7–13.8, *p* < 0.0001)^50^. A subgroup analysis further emphasized the benefit of ^177^Lu-DOTATATE in pNETs, showing a median PFS of 19.4 months versus 8.5 months in the control arm, and an objective response rate (ORR) of 51.2% compared to 12.2%^51^. The NETTER-2 trial demonstrated a higher ORR compared to NETTER-1, despite enrolling patients with higher-grade (Grade 2 and 3) GEP-NETs—a population characterized by more aggressive tumor biology. In contrast, NETTER-1 focused on patients with Grade 1 and 2 midgut NETs, which are generally considered less aggressive. This discrepancy in ORR between the two trials highlights the enhanced efficacy of the PRRT in a treatment naïve population.

The promising outcomes of the NETTER-2 trial underscore the expanding role of PRRT in managing pNETs. Because of this, there has been further exploration of additional PRRT administration beyond the conventional 3–4 dose course^52,53^. Vaughan et al. found that retreatment with PRRT was safe and effective, with a median PFS of 17.5 months (95% CI, 11–23.8) and median OS of 71 months (95% CI, 57-89)^54^. A meta-analysis by Strosberg et al. of seven studies involving 414 patients showed a median pooled PFS of 12.52 months (95% CI, 9.82–15.22) and OS of 26.78 months (95% CI, 18.73–34.83) following PRRT retreatment, with grade 3/4 adverse events (AEs) occurring in 5% of patients, with hematologic events particularly noteworthy^55,56^. Despite the increase in AEs compared to initial PRRT, these findings support PRRT retreatment as a viable option for select patients with tumors that retain SSTR2 expression after initial PRRT treatment^53,56^. Further research is required to address key challenges, including strategies to address hematologic toxicity, and developing standardized protocols for optimal imaging timing for tumor response assessment^57^.

## Combination therapy with ^177^Lu-DOTATATE: enhancing efficacy while managing toxicity

Ongoing research is exploring the potential of combination therapies to enhance therapeutic efficacy of PRRT. Since ^177^Lu-DOTATATE induces DNA damage through radiation, combining it with agents that target DNA repair mechanisms may amplify its therapeutic effects. In addition to the studies highlighted below, numerous other agents are being investigated, as detailed in Table 2.

**Table 2:** Ongoing clinical trials and combination therapies

Clinical trial registry number/phase	Description	Therapeutic Target	Primary Endpoint	Duration	Number Enrolled	Status
NCT03972488/ NETTER-2/ Randomized Phase III	Evaluate the Efficacy and Safety of 177-Lu Dotatate	N/A	PFS	2020–2027	2226	Active
NCT05459844/Randomized Phase III	Comparing 177-Lu Oxodotreotide Injection to Octreotide LAR	N/A	PFS	2022–2028	196	recruiting
NCT05884255/Randomized Phase III	Study of 177Lu-Oxodotreotide Injection	N/A	PFS	2023–2030	220	Not yet recruiting
NCT05247905/ A022001 Phase II Randomized Prospective Trial/	Comparing Capecitabine and Temozolomide in Addition to Lu 177 Dotatate vs Lu 177 Dotatate	Chemotherapeutic agent	PFS	2022–2033	198	Recruiting
NCT04234568/ETCTN 10388/Phase I	Addition of Triapine to 177-Lu Dotatate vs 177-Lu Dotatate	Ribonucleotide reductase inhibitor	MTD & DLT	2020–2024	31	Active,not recruiting
NCT05724108/ETCTN 10558/Randomized Phase II/	Addition of Triapine to Lu 177 Dotatate vs Lu 177 Dotatate	Ribonucleotide reductase inhibitor	ORR	2023–2025	94	Recruiting
NCT04750954/ETCTN 10450/	Addition of Peposertib with 177-Lu Dotatate	DNA dependent protein kinase (DNA-PK) inhibitor	RP2D & DLT	2021–2024	29	Recruiting
NCT05687123/ ETCTN 10479/Phase I	Addition of Sunitinib Malate to 177-Lu Dotatate	Tyrosine kinase inhibitor (activity against VEGF)	AE	2023–2025	24	Recruiting
NCT04086485/ Phase I/II Study	177-Lu Dotatate in Combination With Olaparib	PARP Inhibitor	Phase I: MTD Phase II: ORR	2023–2026	37	Recruiting
NCT03478358/Phase I	Treatment Using Long-lasting Radiolabeled Somatostatin Analog 177-Lu-DOTA-EB-TATE	N/A	Change of SUV of 68Ga-DOTA-TATE & safety of 3.7GBq of 177-Lu-DOTA-EB-TATE with and without amino acid infusion	2017–2023	60	Recruiting
NCT05475210/Phase I Open-Label Study	Safety and Dosimetry of 3-Dose Regimen of Escalating Doses 177-Lu-DOTA-EB-TATE in untreated patients	N/A	Safety & DLT & MTD	2022–2024	9	Recruiting
NCT05477576/ ACTION-1/ phase Ib/III Global, Randomized, Controlled, Open-label Trial	Safety, pharmacokinetics and recommended Phase 3 dose (RP3D) of RYZ101	N/A	Phase Ib: RP3D & Phase III: PFS	2022–2028	218	Recruiting
NCT02609737/ Phase I	Theranostics of Radiolabeled Somatostatin Antagonists 68-Ga-DOTA-JR11 vs177-Lu-DOTA-JR11	N/A	ORR & AE	2015–2020	20	c
NCT04919226/ COMPOSE trial /Randomized, Open-labeled phase III	Efficacy and safety of 177-Lu-edotreotide vs the best standard care	N/A	PFS	2021–2026	202	Recruiting
NCT03049189/COMPETE Trial/ Randomized, Open-label, Multicentre Phase III study	Safety of PRRT With 177-Lu-Edotreotide Compared to Targeted Molecular Therapy With Everolimus	N/A	PFS	2017–2029	309	Active, not recruiting
NCT03590119/ LUTIA/ Randomized Phase II/III trial	Intra-arterial 177-Lu Dotatate adminestration	N/A	Difference in post treatment tumor-to-non-tumor (T/N) activity concentration ratio on SPECT/CT	2018–2022	26	c
NCT04544098/Early phase I clinical trial	Evaluate safety and dosimetry of 177-Lu Dotatate	N/A	number of patients who successfully complete 2 IA injections & ORR	2020–2029	10	Recruiting
NCT04837885/LUTARTERIAL	Intra-arterial Hepatic (IAH) Infusion of Radiolabelled Somatostatin Analogs	N/A	Standardized uptake value (SUVmax) on liver metastases	2021–2024	20	Recruiting

### Combining PRRT with conventional chemotherapy agents

The combination of ^177^Lu-DOTATATE with standard chemotherapeutic agents, such as the antimetabolite capecitabine and the alkylating agent temozolomide (or both, as in the CAPTEM regimen), is being explored as a strategy to improve tumor response^58,59^. Chemotherapeutic agents can inhibit DNA repair pathways, thereby prolonging radiation-induced DNA breaks and increasing apoptosis^60^. Ongoing Phase II trials (NCT027500 and NCT02358356) are investigating the efficacy of these combinations. The E2211 Phase II trial (NCT01824875) provided compelling evidence supporting this approach, demonstrating that CAPTEM significantly improved median PFS to 22.7 months compared to 14.4 months with temozolomide alone (HR = 0.58, *P* = 0.022) in 144 patients. The final analysis showed a median OS of 58.7 months in the CAPTEM arm compared to 53.8 months with temozolomide alone, although the difference was not statistically significant (HR = 0.82, *P* = 0.42)^61^. Additional evidence from the NCT02358356 trial further supports the efficacy of CAPTEM in combination with PRRT. In patients with pNETs, the 12-month PFS was 77% with PRRT + CAPTEM versus 60% in the control arm, suggesting an added benefit of chemotherapy in this subset^62^. However, combining these treatments resulted in a higher incidence of grade 3/4 hematologic toxicities, particularly in patients with midgut NETs, where such events were nearly twice as frequent with PRRT + CAPTEM (88% vs. 46%)^63,64^. To mitigate these risks, careful patient selection, close monitoring, and dose adjustments are essential. Approaches such as prophylactic growth factor support may help reduce the risk of severe neutropenia and thrombocytopenia^65^.

## Combining PRRT with targeted therapy

^177^Lu-DOTATATE exerts its therapeutic effects primarily through β-particle radiation, which causes single-strand DNA breaks in SSTR-positive tumor cells. However, the efficacy of this approach can be limited by intrinsic DNA repair mechanisms that allow tumor cells to recover from radiation-induced damage^66^. Targeting DNA repair proteins such as PARP, HSP90, and checkpoint kinase 1 (CHEK1) holds promise for enhancing the efficacy of radioligand therapy.

### Poly ADP-ribose polymerase (PARP) inhibitors

PARP enzymes play a critical role in DNA repair by addressing single- and double-strand breaks^67^. Inhibiting PARP can enhance the sensitivity of tumor cells to ^177^Lu-DOTATATE by prolonging DNA damage, promoting cell cycle arrest and apoptosis^68–72^. Early results from the Phase I/II study (NCT04086485) combining the PARP inhibitor olaparib with ^177^Lu-DOTATATE indicate the combination is well tolerated with minimal hematologic toxicity, with only grade 1 fatigue and alopecia reported^73,74^. Since PARP inhibitors can also exacerbate bone marrow suppression, close monitoring of hematologic parameters and the use of dose modifications may be necessary^75^. Although PARP inhibitors are FDA-approved for treating pancreatic adenocarcinoma in patients with germline BRCA1/2 mutations, the low incidence of BRCA mutations in pNETS, significantly limits the potential therapeutic benefit of using a mutation-specific patient selection approach^76^.

### DNA repair pathway inhibitors

DNA-PK, a key enzyme in the non-homologous end joining pathway of DNA repair, may prevent the repair of DNA damage caused by ^177^Lu-DOTATATE, thereby enhancing tumor control^77^. Peposertib, a potent radiosensitizer^78^, has been evaluated in combination with radiation and cisplatin chemotherapy in patients with thoracic and head and neck cancers, where it demonstrated acceptable tolerability in early human trials^79^. Maculopapular rash and nausea were the most common Grade 3 AEs^80^. The Experimental Therapeutics Clinical Trials Network (ETCTN) 10450 trial (NCT04750954) is currently evaluating peposertib in combination with ^177^Lu-DOTATATE in patients with well-differentiated, PRRT-naïve GEP-NETs who have progressed on somatostatin analogs. However, this approach may also increase gastrointestinal and hematologic toxicity, highlighting the need for careful patient selection and supportive care measures^81^.

Ribonucleotide reductase, a rate-limiting enzyme in DNA synthesis and repair, can also contribute to resistance against ^177^Lu-DOTATATE by repairing radiation-induced DNA damage. The ETCTN 10388 Phase I trial (NCT04234568) is investigating the combination of the RR inhibitor, Triapine, with ^177^Lu-DOTATATE in SSTR-positive GEP-NETs. Preliminary findings revealed that 22 out of 28 patients remained progression-free at 12 months, indicating the potential of Triapine to overcome PRRT resistance^82^. A Phase II study (ETCTN 10558, NCT05724108) is currently recruiting patients to directly compare the efficacy of Triapine in combination with ^177^Lu-DOTATATE versus ^177^Lu-DOTATATE alone in well-differentiated SSTR-positive NETs^83^.

### Mammalian target of rapamycin (mTOR) inhibitors

The mTOR pathway, which regulates cell growth, metabolism, and DNA damage response, has emerged as a target for enhancing PRRT efficacy^84,85^. Everolimus, an mTOR inhibitor, is FDA approved for the treatment of progressive pNETs, with the RADIANT-3 trial demonstrating a median PFS of 11.0 months compared to 4.6 months with placebo (HR 0.35; *P* < 0.001)^86^. Grade 3 or 4 adverse events (AEs), including anemia (6%) and hyperglycemia (5%), have been reported, with hyperglycemia potentially becoming more severe in patients with preexisting glucose intolerance^86^. The rationale for combining everolimus with ^177^Lu-DOTATATE lies in its ability to inhibit cell proliferation and reduce angiogenesis, which can enhance the cytotoxic effects of radiation^87^. However, conflicting results from preclinical studies also demonstrated increased metastasis in a pancreatic tumor mouse model^88^. Ongoing trials, such as the Phase I/II study (NCT03629847), aim to assess the efficacy of this combination in patients with unresectable Grade 1 and 2 NETs from the gastrointestinal tract, lungs, and pancreas^89^.

### Tyrosine kinase inhibitors

Sunitinib, a tyrosine kinase inhibitor (TKI) targeting multiple enzymes involved in tumor cell proliferation and angiogenesis, improved median PFS (11.4 vs. 5.5 months, HR 0.42, *P* < 0.001) and OS (HR 0.41, *P* = 0.02) in patients with pNETs (NCT00428597)^90^. The rationale for combining sunitinib with ^177^Lu-DOTATATE is based on its ability to enhance the tumor vasculature and oxygenation, possibly increasing radiation sensitivity^91,92^. The study was stopped early due to higher deaths in the placebo group (25% vs. 10%), reinforcing Sunitinib’s benefit^90^. The ongoing ETCTN 10479 Phase I trial (NCT04919226) is assessing the maximum tolerated dose (MTD) of this combination and its impact on AEs^90^.

## Structural modifications of ^177^Lu-DOTATATE

### ^177^Lu-DOTA-EB-TATE: reducing renal toxicity

Efforts to improve the efficacy of ^177^Lu-DOTATATE while minimizing toxicities have led to the development of structural modifications aimed at addressing its limitations. One significant challenge with ^177^Lu-DOTATATE has been its rapid renal cleance, which contributes to increased renal toxicity and reduced tumor retention. To overcome this issue, ^177^Lu-DOTA-EB-TATE, an albumin-binding variant that incorporates Evans blue has been developed. This modification extends the half-life of the radionuclide in the bloodstream, while reducing renal toxicity^93–96^.A Phase I trial (NCT03478358) reported a mPFS of 36 months, an improvement over the 28.4 months observed with conventional ^177^Lu-DOTATATE^97,98^. The toxicity profile was favorable, with no Grade 4 events and limited Grade 3 hematotoxicity (13.3% thrombocytopenia, 3.3% anemia) and Grade 3 hepatotoxicity (3.3%). Notably, no Grade 2/3/4 nephrotoxicity was observed and no cases of leukemia or myelodysplastic syndrome were reported during follow-up^98^. An ongoing Phase I trial (NCT05475210) continues to evaluate its safety and optimal dosage in patients with untreated advanced GEP-NETs^99^.

### ^177^Lu-DOTATOC: an alternative with unique advantages

^177^Lu-DOTATOC, containing the modified somatostatin analog edotreotide, serves as an alternative to ^177^Lu-DOTATATE, which may be particularly valuable during pharmaceutical shortages^64^. A Phase II study investigating ^177^Lu-DOTATOC in patients with GEP-NETs reported a mPFS of 29 months and OS of 47 months^100^. The ORR was 16%, with a disease control rate of 82%^100^. Adverse events were mostly mild (Grade 1 or 2), with Grade 3 or 4 toxicity occurring in less than 10% of patients, primarily hematological, with no Grade 3 or 4 renal toxicity^101^.The ongoing Phase III COMPOSE trial (NCT04919226) aims to compare ^177^Lu-DOTATOC to standard care options such as CAPTEM, everolimus, and FOLFOX in patients with aggressive Grade 2/3 SSTR-positive GEP-NETs^102^. Results from this trial will be critical in determining cost-effectiveness and clinical utility, especially given the lower production costs and greater availability of ^177^Lu-DOTATOC compared to ^177^Lu-DOTATATE^64^.The Phase III COMPETE trial (NCT03049189), is evaluating ^177^Lu-DOTATOC versus everolimus in SSTR-positive GEP-NETs, and has met its primary objective of prolonging median PFS to 23.9 months compared to 14.1 months on everolimus (*p* value = 0.022)^103^. While OS data is still maturing, the trial marks the first instance of a targeted radiopharmaceutical therapy outperforming a molecular therapy in this setting, with plans for a U.S. New Drug Application submission in 2025^104,105^.

## Targeted alpha therapy

**Fig. 2:**
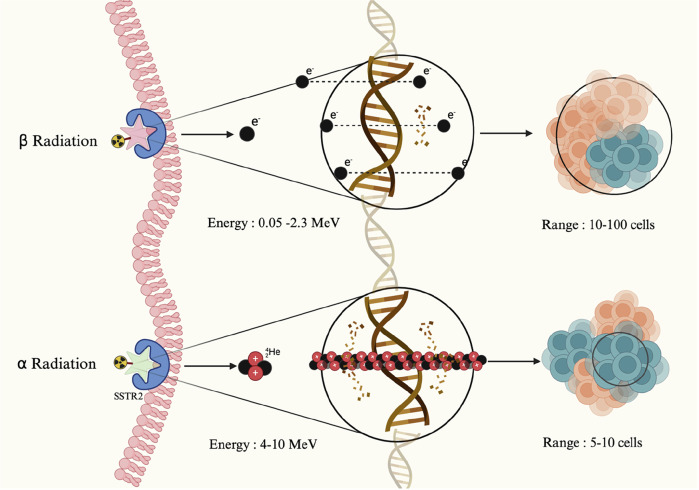
Beta and alpha radiation in targeted radionuclide therapy. This figure illustrates the mechanisms of targeted radionuclide therapy via somatostatin receptor type 2 (SSTR2). The top pathway depicts beta radiation, which affects a broader area, targeting 10-100 cells and causing single-stranded DNA damage through the emission of electrons with a longer range. In contrast, the bottom pathway highlights alpha radiation, which impacts a much smaller area, targeting only 5-10 cells. Alpha particles deliver higher energy with a shorter range, resulting in highly localized and concentrated DNA damage, leading to irreparable double-strand breaks in tumor cells. Created in Biorender Karimi, A. (2025).

Targeted Alpha Therapy (TAT) presents a compelling alternative to beta-emitting PRRT by utilizing alpha particles, that cause highly localized, irreparable DNA double-strand breaks due to their large size and short travel range than beta particles^106^. In contrast, beta emitters predominantly induce single-strand DNA breaks, that are more easily repaired, potentially contributing to tumor resistance^107^. Due to their limited tissue penetration, alpha particles provide precise tumor targeting with minimal off-target effects^108,109^, makes TAT particularly attractive for treating liver metastases (Fig. 2)^110^. Although preliminary results are encouraging, TAT remains in the early stages of clinical development^111^. Ongoing trials are exploring the safety and efficacy of these agents (Table 3).

**Table 3:** Ongoing TAT trials

Clinical trial registry number/phase	Radiopharmaceutical	Number enrolled	Duration	Primary endpoint
NCT05153772/Phase II trial	^212^Pb-DOTAMTATE	69	2021–2026	Assess Safety and efficiency
NCT05636618/phase 1/2a	^212^Pb-VMT-α-NET	10	2022–2027	Assess safety & RP2D
NCT05477576/ACTION-1/Phase 3/Randomized Open-label	^225^Ac -DOTATATE (RYZ101)	288		TEAEs & AE

### 213-bismuth (^213^Bi)

^213^Bi-DOTATOC has shown considerable promise in preclinical studies^112^, significantly reducing tumor growth without causing chronic kidney damage or hematologic toxicity^113^. In a Phase I trial involving seven patients with pNETs that progressed after ^177^Lu-DOTATOC therapy, ^213^Bi-DOTATOC induced sustained tumor responses with lower acute hematotoxicity and moderate chronic kidney toxicity, suggesting it may offer a favorable safety profile for tumors resistant to beta radiation^114^. In a related clinical study, Giesel et al. demonstrated that contrast-enhanced ultrasound is a reliable modality for assessing treatment response in patients treated with ^213^Bi-DOTATOC by detecting early changes in tumor microcirculation perfusion^115^. The study found that 66% of patients treated with ^213^Bi-DOTATOC showed a significant reduction in enhancement, with more pronounced declines observed during short-term follow-up, compared to 33% of patients treated with ^177^Lu/^90^Y-DOTATOC^115^.

### 225-actinium (^225^Ac)

^225^Ac, an alpha-emitter with a longer half-life of 9.9 days, has become a focal point in TAT research. Preclinical studies in mouse models of liver metastases from pancreatic NETs demonstrated that ^225^Ac-DOTATOC significantly improved survival compared to non-radioactive DOTATOC^116^. In a study by Ballal et al., patients who had achieved disease control with prior ^177^Lu-DOTATOC therapy and were subsequently retreated with ^225^Ac-DOTATATE showed the best outcomes, with a 24-month OS rate of 95%^117^. The ongoing ACTION-1 Phase III clinical trial (NCT05477576) is evaluating ^225^Ac -DOTATATE in patients with well-differentiated GEP-NETs that have progressed following ^177^Lu-DOTATATE therapy^118^. Preliminary data from the Phase 1b portion of the trial reported promising efficacy, with an ORR of 29.4%, including a complete response in one patient, partial responses in four patientsand stable disease in 41.2% of participants. While the median PFS was not yet estimable, early results suggest durable responses^119^. A notable case report further demonstrated the potential of ^225^Ac in tandem-PRRT approaches. A patient with rapidly progressing pNET and extensive metastases demonstrated an exceptional response to tandem-PRRT using ^177^Lu-DOTA-LM3 and ^225^Ac-DOTA-LM3 after exhausting prior chemotherapy and ^177^Lu-DOTATATE PRRT options^120^. This combination therapy led to significant improvements across all metastatic sites, particularly in the liver, highlighting the synergistic effects of alpha and beta emitters^120^.

### 212-lead (^212^Pb)

^212^Pb is another alpha-emitter under investigation in combination with various chelating agents. In a study by Delpasand et al, GEP-NET patients receiving ^212^Pb-DOTAMTATE exhibited an 80% objective radiologic response, with the treatment being well tolerated and no severe AEs reported^121^. The ongoing ALPHAMEDIX02 Phase II trial (NCT05153772) is investigating ²¹²Pb-DOTAMTATE in SSTR-positive, PRRT-naïve NET patients. Pooled results from Phase I/II trials demonstrated a high ORR of 56.8%, with the median response duration of 14 months (range: 5–22 months), indicating a promising efficacy profile^122^. Additionally, a Phase I/IIa study (NCT05636618) is evaluating ^212^Pb VMT-alpha-NET, which employs a novel polyethylene linker to conjugate octreotide to ^212^Pb for advanced SSTR2-positive, PRRT-naïve NET patients^123^. In this small cohort, 9 out of 10 patients (90%) who completed all treatment cycles remained progression-free at the last follow-up, with a median follow-up duration of 17.4 months (range: 9–26 months)^124^. Common AEs included nausea (31%) and alopecia (25%), while Grade 3 toxicities occurred in 5% of patients, with no reported Grade 4 events^124^.

While TAT offers significant advantages over beta-emitting PRRT—including higher linear energy transfer, superior tumor control, and the ability to eradicate targeted metastatic lesions—its widespread clinical application faces substantial challenges^125,126^. Radionuclide availability and high production costs is a major hurdle, as alpha emitters require complex production processes and are limited to a few specialized facilities worldwide^127^. From a logistical perspective, the short half-lives of many alpha emitters complicate transport, storage, and coordination between production sites and clinical centers^128^. Moreover, the short path length of alpha particles necessitates precise targeting to minimize off-target effects, requiring advanced imaging techniques to ensure optimal therapeutic index^129,130^. To overcome these barriers, strategies such as streamlined cost-effective production processes and establishing international guidelines for the safe and efficient use of TAT will be crucial for its integration into standard clinical practice^130^.

## SSTR2 antagonists: advancing imaging and therapy

Historically, it was believed that internalization of radiotracers was necessary for effective SSTR-targeted therapy and imaging. However, research by Ginj et al. challenged this paradigm by demonstrating that SSTR2 antagonists could offer superior efficacy compared to agonists^131^. Antagonists bind to a greater number of receptor sites on the tumor surface without internalizing, thereby providing higher binding affinity and specificity^131,132^. Several SSTR2 antagonists have been developed and tested in preclinical and clinical studies.

### ^177^Lu-DOTA-JR11

^177^Lu-DOTA-JR11, an SSTR2 antagonist, has emerged as a leading candidate for clinical translation due to its ability to deliver high radiation doses selectively to SSTR-positive tumors^29^. Its companion imaging agent, ^68^Ga-NODAGA-JR11, has demonstrated superior diagnostic accuracy compared to ^68^Ga-DOTATATE^133,134^. In two human studies, ^68^Ga-NODAGA-JR11 outperformed ^68^Ga-DOTATATE in sensitivity (91.7% vs. 77.2%) and lesion detection (1095 vs. 1003 lesions, *P* = 0.007), providing better image contrast, particularly in patients with low to intermediate-grade GEP-NETs. Moreover, ^68^Ga-NODAGA-JR11 exhibited a significantly higher target-to-background ratio in liver lesions (6.4 ± 8.7 vs. 3.1 ± 2.6, *P* = 0.000), thereby enhancing its diagnostic accuracy and potentially enabling more accurate staging and treatment planning^133,134^.

#### LM3,4

SSTR2 antagonists LM3 and LM4 have demonstrated significant potential in both imaging and therapeutic applications. In clinical settings, 177Lu-DOTA-LM3 PRRT achieved a disease control rate of 85.1%, with 36.2% of patients experiencing a partial response^135^. The treatment was well tolerated, with only mild nausea (9.8%) and thrombocytopenia (5.9%) reported, and no cases of severe nephrotoxicity, hepatotoxicity, or hematologic toxicity^135^. In diagnostic applications, ^68^Ga-NODAGA-LM3 and ^68^Ga-DOTA-LM3 have demonstrated significant accuracy for NET detection compared to ^68^Ga-DOTATATE^136^. ^68^Ga-NODAGA-LM3 showed significantly higher tumor uptake (SUVmax: 29.1 vs. 21.6, *P* < 0.05) and an improved tumor-to-liver ratio (5.0 vs. 2.9, *P* < 0.05), thereby enhancing its diagnostic accuracy. Similarly, ^68^Ga-DOTA-LM3 demonstrated a higher tumor-to-liver ratio (5.2 vs. 2.1, *P* < 0.05) while maintaining lower uptake in normal organs, thus improving image contrast and lesion detection^136^. LM4, a modified version of LM3, has shown enhanced tumor retention and reduced kidney uptake in preclinical studies^137^. In human studies, ^68^Ga-DATA5m-LM4 demonstrated high tumor uptake with SUVmax reaching 167.93 (mean ± SD: 44.47 ± 36.22). When compared directly to ^68^Ga-DOTA-TATE, ^68^Ga-DATA5m-LM4 exhibited significantly lower uptake in normal liver parenchyma (SUVmean: 3.90 ± 0.88 vs. 9.12 ± 3.64, *P* < 0.000001) as well as in the thyroid, pancreas, and spleen (*P* < 0.05). This favorable biodistribution, characterized by high tumor contrast and minimal background uptake, underscores the potential of ^68^Ga-DATA5m-LM4 as an enhanced imaging agent for NET staging^138^.

##### Challenges and future directions

Theranostics has significantly transformed the management of pNETs by integrating molecular imaging with targeted therapy, thereby enhancing both diagnostic precision and therapeutic efficacy. Beta-emitting PRRT, particularly ^177^Lu-DOTATATE, is becoming a cornerstone treatment with positive results in both first- and second-line settings. However, the limited response rates and development of treatment resistance have highlighted the urgent need for alternative strategies. In this review, we have explored combination approaches involving other systemic therapies as well as emerging radiopharmaceuticals including TATs and SSTR2 antagonists. While these innovative therapies offer substantial potential, their widespread clinical implementation faces several barriers, including combined toxicities, challenges in radionuclide production and transport, high costs, and the necessity for specialized infrastructure.

Biomarker-driven patient selection represents a critical area for future research to ensure that theranostic approaches are targeted to the most suitable candidates. Currently, patient selection is primarily based on the intensity of SSTR expression assessed through PET imaging and the exclusion of patients with preexisting glucose intolerance, anemia, thrombocytopenia, or renal disease. However, emerging blood-based biomarkers offer promising alternatives for predicting PRRT sensitivity and treatment efficacy. For instance, the NETest, a 51-multigene assay utilizing PCR analysis of specific NET circulating transcripts, generates a score reflecting real-time tumor activity and has shown potential in predicting PRRT outcomes^130,139^. Similarly, an inflammation-based index, derived from serum C-reactive protein and albumin levels is being investigated as a prognostic tool for assessing survival and treatment response in patients with metastatic NETs^140^. The development of such biomarkers could significantly enhance patient stratification.

Another critical challenge that demands attention is the long-term safety of these therapies, particularly regarding hematologic and renal toxicity^48,141^. The cumulative effects of radiation-induced toxicity remain inadequately explored, necessitating prolonged follow-up studies and the development of risk mitigation strategies^57^. Advances in personalized dosimetry could play a pivotal role in this regard by allowing for individualized radiation doses that maximize therapeutic benefits while minimizing toxicity risks^97^. Optimizing dosimetric approaches could enable the safe administration of higher radiation doses to patients with aggressive tumor phenotypes or poor prognostic indicators .

In conclusion, the continued integration of molecular imaging and novel radiopharmaceuticals holds the potential to advance pNET treatment by creating personalized therapeutic strategies. The path forward will require a multidisciplinary approach, but the promising clinical outcomes to date underscore the transformative potential of these innovative theranostic strategies.

## Data Availability

No datasets were generated or analysed during the current study.

## References

[1] McKenna, L. R. & Edil, B. H. Update on pancreatic neuroendocrine tumors. *Gland Surg.* **3**, 258–275 (2014). 25493258 10.3978/j.issn.2227-684X.2014.06.03 PMC4244504

[2] Hill, J. S. et al. Pancreatic neuroendocrine tumors: The impact of surgical resection on survival. *Cancer* **115**, 741–751 (2009). 19130464 10.1002/cncr.24065

[3] Bilimoria, K. Y. et al. Clinicopathologic features and treatment trends of pancreatic neuroendocrine tumors: analysis of 9,821 patients. *J. Gastrointest. Surg.* **11**, 1460–1467 (2007). Novdiscussion 1467-9. 17846854 10.1007/s11605-007-0263-3

[4] Pathak, S., Starr, J. S., Halfdanarson, T., Sonbol, M. B. Understanding the increasing incidence of neuroendocrine tumors. 10.1080/17446651.2023.2237593 (2023). 10.1080/17446651.2023.2237593 37466336

[5] Cloyd, J. M. Poultsides GA. Non-functional neuroendocrine tumors of the pancreas: Advances in diagnosis and management. *World J. Gastroenterol.* **21**, 9512–9525 (2015). 26327759 10.3748/wjg.v21.i32.9512 PMC4548112

[6] Liaquat M. T., Kasi A. Pancreatic Islet Cell Cancer. *StatPearls*. StatPearls Publishing. Copyright © 2023, StatPearls Publishing LLC.; (2023). 32809656

[7] Ampofo, E., Nalbach, L., Menger, M. D. & Laschke, M. W. Regulatory mechanisms of somatostatin expression. *Int. J. Mol. Sci.* **21**, 4170 (2020). 32545257 10.3390/ijms21114170 PMC7312888

[8] Qian, Z. R. et al. Association between somatostatin receptor expression and clinical outcomes in neuroendocrine tumors. *Pancreas* **45**, 1386–1393 (2016). 27622342 10.1097/MPA.0000000000000700 PMC5067972

[9] Zhu, L. M. et al. Differences and similarities in the clinicopathological features of pancreatic neuroendocrine tumors in China and the United States: A multicenter study. *Med. (Baltim.)* **95**, e2836 (2016). 10.1097/MD.0000000000002836 PMC4998644 26886644

[10] Kaemmerer, D. et al. Inverse expression of somatostatin and CXCR4 chemokine receptors in gastroenteropancreatic neuroendocrine neoplasms of different malignancy. *Oncotarget* **6**, 27566–27579 (2015). 26259237 10.18632/oncotarget.4491 PMC4695009

[11] Gomes-Porras M., Cárdenas-Salas, J., Álvarez-Escolá, C. Somatostatin analogs in clinical practice: A review. *Int. J. Mol. Sci*. 29;21 10.3390/ijms21051682 (2020). 10.3390/ijms21051682 PMC7084228 32121432

[12] Mizutani, G. et al. Expression of somatostatin receptor (SSTR) subtypes (SSTR-1, 2A, 3, 4 and 5) in neuroendocrine tumors using real-time RT-PCR method and immunohistochemistry. *Acta Histochem Cytochem* **45**, 167–176 (2012). 22829710 10.1267/ahc.12006 PMC3395302

[13] Sanli, Y. et al. Neuroendocrine tumor diagnosis and management: 68Ga-DOTATATE PET/CT. *Am. J. Roentgenol.* **211**, 267–277 (2018). 29975116 10.2214/AJR.18.19881

[14] Khanna, L. et al. Pancreatic neuroendocrine neoplasms: 2020 update on pathologic and imaging findings and classification. *Radiographics* **40**, 1240–1262 (2020). 32795239 10.1148/rg.2020200025

[15] Hu, Y. et al. Role of somatostatin receptor in pancreatic neuroendocrine tumor development, diagnosis, and therapy. *Front Endocrinol. (Lausanne)* **12**, 679000 (2021). 34093445 10.3389/fendo.2021.679000 PMC8170475

[16] Lee, L., Ito, T. & Jensen, R. T. Imaging of pancreatic neuroendocrine tumors: Recent advances, current status, and controversies. *Expert Rev. Anticancer Ther.* **18**, 837–860 (2018). 29973077 10.1080/14737140.2018.1496822 PMC6283410

[17] Krebs, S. et al. Biodistribution and radiation dose estimates for 68Ga-DOTA-JR11 in patients with metastatic neuroendocrine tumors. *Eur. J. Nucl. Med. Mol. Imaging* **46**, 677–685 (2019). 30374529 10.1007/s00259-018-4193-y PMC6447060

[18] Walker, R. C. et al. Measured human dosimetry of68Ga-DOTATATE. *J. Nucl. Med.* **54**, 855–860 (2013). 23516312 10.2967/jnumed.112.114165 PMC4472480

[19] Lee, I. et al. Comparison of diagnostic sensitivity and quantitative indices between 68Ga-DOTATOC PET/CT and 111In-pentetreotide SPECT/CT in neuroendocrine tumors: A preliminary report. *Nucl. Med. Mol. Imaging* **49**, 284–290 (2015). 26550047 10.1007/s13139-015-0356-y PMC4630328

[20] Deppen, S. A. et al. Safety and efficacy of 68Ga-DOTATATE PET/CT for diagnosis, staging, and treatment management of neuroendocrine tumors. *J. Nucl. Med.* **57**, 708–714 (2016). 26769865 10.2967/jnumed.115.163865 PMC5362940

[21] Malan, N., & Vangu, M.-D.-T. Normal variants, pitfalls and artifacts in Ga-68 DOTATATE PET/CT imaging. *Front. Nuclear Med*. 2022;2 10.3389/fnume.2022.825486 (2022). 10.3389/fnume.2022.825486 PMC11440971 39354987

[22] Poeppel, T. D. et al. 68Ga-DOTATOC versus 68Ga-DOTATATE PET/CT in functional imaging of neuroendocrine tumors. *J. Nucl. Med.* **52**, 1864–1870 (2011). 22072704 10.2967/jnumed.111.091165

[23] Deroose, C. M. et al. Molecular imaging of gastroenteropancreatic neuroendocrine tumors: Current status and future directions. *J. Nucl. Med* **57**, 1949–1956 (2016). 27811124 10.2967/jnumed.116.179234 PMC6952053

[24] Pfeifer, A. et al. 64Cu-DOTATATE PET for neuroendocrine tumors: A prospective head-to-head comparison with 111In-DTPA-Octreotide in 112 patients. *J. Nucl. Med* **56**, 847–854 (2015). 25952736 10.2967/jnumed.115.156539

[25] Delpassand, E. S. et al. (64)Cu-DOTATATE PET/CT for imaging patients with known or suspected somatostatin receptor-positive neuroendocrine tumors: Results of the first U.S. prospective, reader-masked clinical trial. *J. Nucl. Med.* 61, 890–896 (2020). 10.2967/jnumed.119.236091 31924723

[26] Johnbeck, C. B., Knigge, U. & Kjær, A. PET tracers for somatostatin receptor imaging of neuroendocrine tumors: Current status and review of the literature. *Future Oncol.* **10**, 2259–2277 (2014). 25471038 10.2217/fon.14.139

[27] Leupe, H. et al. (18)F-labeled somatostatin analogs as PET tracers for the somatostatin receptor: Ready for clinical use. *J. Nucl. Med*. **64**, 835–841 (2023). 37169533 10.2967/jnumed.123.265622

[28] Johnbeck, C. B. et al. Head-to-head comparison of (64)Cu-DOTATATE and (68)Ga-DOTATOC PET/CT: A prospective study of 59 patients with neuroendocrine tumors. *J. Nucl. Med.* **58**, 451–457 (2017). 27660147 10.2967/jnumed.116.180430

[29] Fani, M., Nicolas, G. P. & Wild, D. Somatostatin receptor antagonists for imaging and therapy. *J. Nucl. Med* **58**, 61s–66s (2017). 28864614 10.2967/jnumed.116.186783

[30] Zhu, W. et al. Head-to-head comparison of 68Ga-DOTA-JR11 and 68Ga-DOTATATE PET/CT in patients with metastatic, well-differentiated neuroendocrine tumors: A prospective study. *J. Nucl. Med.* **61**, 897–903 (2020). 31676731 10.2967/jnumed.119.235093 PMC7262225

[31] Majala, S. et al. Prediction of the aggressiveness of non-functional pancreatic neuroendocrine tumors based on the dual-tracer PET/CT. *EJNMMI Res.* **9**, 116 (2019). 31872324 10.1186/s13550-019-0585-7 PMC6928175

[32] Paiella, S. et al. Dual-Tracer (68Ga-DOTATOC and 18F-FDG-)-PET/CT Scan and G1-G2 nonfunctioning pancreatic neuroendocrine tumors: A single-center retrospective evaluation of 124 nonmetastatic resected cases. *Neuroendocrinology* **112**, 143–152 (2022). 33508821 10.1159/000514809

[33] Mapelli, P. et al. Dual Tracer 68Ga-DOTATOC and 18F-FDG PET improve preoperative evaluation of aggressiveness in resectable pancreatic neuroendocrine neoplasms. *Diagnostics (Basel)*. 28;11 10.3390/diagnostics11020192 (2021). 10.3390/diagnostics11020192 PMC7912034 33525712

[34] Mapelli, P. et al. <strong>Dual tracer 68Ga-DOTATOC and 18F-FDG PET/CT for preoperative risk evaluation in pancreatic neuroendocrine tumours: explorative texture analysis</strong>. *J. Nucl. Med.* **60**, 476–476 (2019).

[35] Zhou, Y. et al. Heterogeneous Uptake of 68 Ga-DOTATATE and 18 F-FDG in initial diagnosed neuroendocrine tumors patients : Which patients are suitable for dual-tracer PET imaging? *Clin. Nucl. Med* **49**, 516–520 (2024). 38637950 10.1097/RLU.0000000000005231

[36] Gao, J., Xu, S., Ju, H., Pan, Y. & Zhang, Y. The potential application of MR-derived ADCmin values from 68Ga-DOTATATE and 18F-FDG dual tracer PET/MR as replacements for FDG PET in assessment of grade and stage of pancreatic neuroendocrine tumors. *EJNMMI Res.* **13**, 10 (2023). 36752942 10.1186/s13550-023-00960-z PMC9908795

[37] Jawlakh, H., Velikyan, I., Welin, S. & Sundin, A. (68) Ga-DOTATOC-PET/MRI and (11) C-5-HTP-PET/MRI are superior to (68) Ga-DOTATOC-PET/CT for neuroendocrine tumour imaging. *J Neuroendocrinol* **33**, e12981 (2021). 10.1111/jne.12981 34046974

[38] Halfdanarson, T. R., Rabe, K. G., Rubin, J. & Petersen, G. M. Pancreatic neuroendocrine tumors (PNETs): Incidence, prognosis and recent trend toward improved survival. *Ann. Oncol.* **19**, 1727–1733 (2008). 18515795 10.1093/annonc/mdn351 PMC2735065

[39] Ma, Z. Y. et al. Pancreatic neuroendocrine tumors: A review of serum biomarkers, staging, and management. *World J. Gastroenterol.* **26**, 2305–2322 (2020). 32476795 10.3748/wjg.v26.i19.2305 PMC7243647

[40] Ito, T. et al. JNETS clinical practice guidelines for gastroenteropancreatic neuroendocrine neoplasms: Diagnosis, treatment, and follow-up: a synopsis. *J. Gastroenterol.* **56**, 1033–1044 (2021). 34586495 10.1007/s00535-021-01827-7 PMC8531106

[41] Rinke, A. et al. Placebo-controlled, double-blind, prospective, randomized study on the effect of octreotide LAR in the control of tumor growth in patients with metastatic neuroendocrine midgut tumors (PROMID): Results of long-term survival. *Neuroendocrinology* **104**, 26–32 (2017). 26731483 10.1159/000443612

[42] Caplin, M. E. et al. Lanreotide autogel/depot in advanced enteropancreatic neuroendocrine tumours: Final results of the CLARINET open-label extension study. *Endocrine* **71**, 502–513 (2021). 33052555 10.1007/s12020-020-02475-2 PMC7881960

[43] Caplin, M. E. et al. Lanreotide in metastatic enteropancreatic neuroendocrine tumors. *N. Engl. J. Med*. **371**, 224–233 (2014). 25014687 10.1056/NEJMoa1316158

[44] Das, S., Al-Toubah, T., El-Haddad, G. & Strosberg, J. (177)Lu-DOTATATE for the treatment of gastroenteropancreatic neuroendocrine tumors. *Expert Rev. Gastroenterol. Hepatol.* **13**, 1023–1031 (2019). 31652074 10.1080/17474124.2019.1685381 PMC7227421

[45] Strosberg, J. R. et al. 177Lu-Dotatate plus long-acting octreotide versus high‑dose long-acting octreotide in patients with midgut neuroendocrine tumours (NETTER-1): final overall survival and long-term safety results from an open-label, randomised, controlled, phase 3 trial. *Lancet Oncol.* **22**, 1752–1763 (2021). 34793718 10.1016/S1470-2045(21)00572-6

[46] Strosberg, J. R. et al. (177)Lu-Dotatate plus long-acting octreotide versus high‑dose long-acting octreotide in patients with midgut neuroendocrine tumours (NETTER-1): final overall survival and long-term safety results from an open-label, randomised, controlled, phase 3 trial. *Lancet Oncol.* **22**, 1752–1763 (2021). 34793718 10.1016/S1470-2045(21)00572-6

[47] (FDA) FaDA. FDA approves lutetium Lu 177 dotatate for treatment of GEP-NETS. Accessed 01/26/2018, 2018. https://www.fda.gov/drugs/resources-information-approved-drugs/fda-approves-lutetium-lu-177-dotatate-treatment-gep-nets.

[48] Alsadik, S. et al. Safety of peptide receptor radionuclide therapy with (177)Lu-DOTATATE in neuroendocrine tumor patients with chronic kidney disease. *J. Nucl. Med* **63**, 1503–1508 (2022). 35210299 10.2967/jnumed.121.263056 PMC9536708

[49] Brabander, T. et al. Long-term efficacy, survival, and safety of [(177)Lu-DOTA(0),Tyr(3)]octreotate in patients with gastroenteropancreatic and bronchial neuroendocrine tumors. *Clin. Cancer Res*. **23**, 4617–4624 (2017). 10.1158/1078-0432.CCR-16-2743 28428192

[50] Singh, S. et al. [177Lu]Lu-DOTA-TATE plus long-acting octreotide versus high‑dose long-acting octreotide for the treatment of newly diagnosed, advanced grade 2–3, well-differentiated, gastroenteropancreatic neuroendocrine tumours (NETTER-2): An open-label, randomised, phase 3 study. *Lancet* **403**, 2807–2817 (2024). 38851203 10.1016/S0140-6736(24)00701-3

[51] Scott, R. Lutetium Lu 177 dotatate maintains PFS, ORR benefits across subgroups in SSTR+ GEP-NETs. Onclive. Accessed 6/26, 2024. https://www.onclive.com/view/lutetium-lu-177-dotatate-maintains-pfs-orr-benefits-across-subgroups-in-sstr-gep-nets.

[52] Strosberg, J. et al. Phase 3 trial of (177)Lu-dotatate for midgut neuroendocrine tumors. *N. Engl. J. Med.* **376**, 125–135 (2017). 28076709 10.1056/NEJMoa1607427 PMC5895095

[53] Rodrigues, M. et al. Long-term survival and value of (18)F-FDG PET/CT in patients with gastroenteropancreatic neuroendocrine tumors treated with second peptide receptor radionuclide therapy course with (177)Lu-DOTATATE. *Life (Basel)*. 4;11 10.3390/life11030198 (2021). 10.3390/life11030198 PMC8000415 33806393

[54] Vaughan, E. et al. Retreatment with peptide receptor radionuclide therapy in patients with progressing neuroendocrine tumours: Efficacy and prognostic factors for response. *Br. J. Radiol.* **91**, 20180041 (2018). 29513039 10.1259/bjr.20180041 PMC6475926

[55] Strosberg, J., Leeuwenkamp, O. & Siddiqui, M. K. Peptide receptor radiotherapy re-treatment in patients with progressive neuroendocrine tumors: A systematic review and meta-analysis. *Cancer Treat. Rev.* **93**, 102141 (2021). 33418096 10.1016/j.ctrv.2020.102141

[56] Puliani, G. et al. New insights in PRRT: Lessons From 2021. Mini review. *Front. Endocrinol*. 2022;13 10.3389/fendo.2022.861434 (2022). 10.3389/fendo.2022.861434 PMC9016202 35450421

[57] Liberini, V. et al. The challenge of evaluating response to peptide receptor radionuclide therapy in gastroenteropancreatic neuroendocrine tumors: The present and the future. *Diagnostics* **10**, 1083 (2020). 33322819 10.3390/diagnostics10121083 PMC7763988

[58] Ballal, S., Yadav, M. P., Damle, N. A., Sahoo, R. K. & Bal, C. Concomitant 177Lu-DOTATATE and capecitabine therapy in patients with advanced neuroendocrine tumors: A long-term-outcome, toxicity, survival, and quality-of-life study. *Clin. Nucl. Med*. **42**, e457–e466 (2017). 28872545 10.1097/RLU.0000000000001816

[59] van Essen, M. et al. Report on short-term side effects of treatments with 177Lu-octreotate in combination with capecitabine in seven patients with gastroenteropancreatic neuroendocrine tumours. *Eur. J. Nucl. Med. Mol. Imaging* **35**, 743–748 (2008). 18188559 10.1007/s00259-007-0688-7 PMC2668587

[60] Li, L. Y., Guan, Y. D., Chen, X. S., Yang, J. M. & Cheng, Y. DNA repair pathways in cancer therapy and resistance. *Front Pharm.* **11**, 629266 (2020). 10.3389/fphar.2020.629266 PMC7898236 33628188

[61] Kunz, P. L. et al. Randomized study of temozolomide or temozolomide and capecitabine in patients with advanced pancreatic neuroendocrine tumors (ECOG-ACRIN E2211). *J. Clin. Oncol.* **41**, 1359–1369 (2023). 36260828 10.1200/JCO.22.01013 PMC9995105

[62] Pavlakis N. Capecitabine ON temozolomide radionuclide therapy octreotate lutetium-177 NeuroEndocrine Tumours Study (CONTROL NETS). clinicaltrials.gov. Accessed 5/7, 2022. https://clinicaltrials.gov/study/NCT02358356.

[63] Group AG-IT. Capecitabine ON Temozolomide Radionuclide Therapy Octreotate Lutetium-177 NeuroEndocrine Tumours Study. My Cancer Genome. 2017. Accessed 11/22/2017, 2017. https://clinicaltrials.gov/show/NCT02358356

[64] https://clin.larvol.com/trial-detail/NCT02358356.

[65] Yordanova A., & Ahmadzadehfar, H. Combination therapies with PRRT. *Pharmaceuticals (Basel)*. 30;14 10.3390/ph14101005 (2021). 10.3390/ph14101005 PMC8538931 34681229

[66] O’Neill, E. et al. Imaging DNA damage repair in vivo after (177)Lu-DOTATATE therapy. *J. Nucl. Med* **61**, 743–750 (2020). 31757844 10.2967/jnumed.119.232934 PMC7198382

[67] Ray Chaudhuri, A. & Nussenzweig, A. The multifaceted roles of PARP1 in DNA repair and chromatin remodelling. *Nat. Rev. Mol. Cell Biol.* **18**, 610–621 (2017). 28676700 10.1038/nrm.2017.53 PMC6591728

[68] Camero, S. et al. PARP inhibitors affect growth, survival and radiation susceptibility of human alveolar and embryonal rhabdomyosarcoma cell lines. *J. Cancer Res Clin. Oncol.* **145**, 137–152 (2019). 30357520 10.1007/s00432-018-2774-6 PMC6326011

[69] Lesueur, P. et al. Poly-(ADP-ribose)-polymerase inhibitors as radiosensitizers: a systematic review of pre-clinical and clinical human studies. *Oncotarget* **8**, 69105–69124 (2017). 28978184 10.18632/oncotarget.19079 PMC5620324

[70] Purohit, N. K. et al. Potentiation of (177)Lu-octreotate peptide receptor radionuclide therapy of human neuroendocrine tumor cells by PARP inhibitor. *Oncotarget* **9**, 24693–24706 (2018). 29872498 10.18632/oncotarget.25266 PMC5973847

[71] Nonnekens, J. et al. Potentiation of peptide receptor radionuclide therapy by the PARP inhibitor olaparib. *Theranostics* **6**, 1821–1832 (2016). 27570553 10.7150/thno.15311 PMC4997239

[72] Cullinane, C. et al. Enhancing the anti-tumour activity of (177)Lu-DOTA-octreotate radionuclide therapy in somatostatin receptor-2 expressing tumour models by targeting PARP. *Sci. Rep.* **10**, 10196 (2020). 32576907 10.1038/s41598-020-67199-9 PMC7311440

[73] Lin, F. et al. <strong>Phase 2 study of Lu-177-DOTATATE in combination with olaparib in patients with metastatic or inoperable GI neuroendocrine tumors - first results on safety and efficacy</strong>. *J. Nucl. Med.* **64**, P1299–P1299 (2023). (supplement 1).

[74] Hallqvist, A. et al. Optimizing the Schedule of PARP Inhibitors in Combination with (177)Lu-DOTATATE: A dosimetry rationale. *Biomedicines*. Oct 29;9 10.3390/biomedicines9111570 (2021). 10.3390/biomedicines9111570 PMC8615768 34829796

[75] Zhou, J. X., Feng, L. J. & Zhang, X. Risk of severe hematologic toxicities in cancer patients treated with PARP inhibitors: A meta-analysis of randomized controlled trials. *Drug Des. Devel Ther.* **11**, 3009–3017 (2017). 29075104 10.2147/DDDT.S147726 PMC5648323

[76] Mohindroo, C. et al. Prevalence of germline variants in patients with pancreatic neuroendocrine tumors. *J. Clin. Oncol.* **41**, 4135–4135 (2023).

[77] Chauhan, A. et al. ETCTN 10450: A phase I trial of peposertib and lutetium 177 DOTATATE in well-differentiated somatostatin receptor-positive gastroenteropancreatic neuroendocrine tumors (GEP-NETs). *J. Clin. Oncol.* **41**, TPS658–TPS658 (2023).

[78] Romesser, P. B. et al. A phase Ib study of the DNA-PK inhibitor peposertib combined with neoadjuvant chemoradiation in patients with locally advanced rectal cancer. *Clin. Cancer Res.* **30**, 695–702 (2024). 38051750 10.1158/1078-0432.CCR-23-1129 PMC10870114

[79] Samuels, M. et al. A phase 1 study of the DNA-PK inhibitor peposertib in combination with radiation therapy with or without cisplatin in patients with advanced head and neck tumors. *Int. J. Radiat. Oncol. Biol. Phys.* **118**, 743–756 (2024). 37751793 10.1016/j.ijrobp.2023.09.024

[80] van Bussel, M. T. J. et al. A first-in-man phase 1 study of the DNA-dependent protein kinase inhibitor peposertib (formerly M3814) in patients with advanced solid tumours. *Br. J. Cancer* **124**, 728–735 (2021). 33230210 10.1038/s41416-020-01151-6 PMC7884679

[81] Testing the Addition of An Anti-cancer Drug, M3814 (Peposertib), to the Usual Radiation-Based Treatment (Lutetium Lu 177 Dotatate) for Pancreatic Neuroendocrine Tumors. Clinicaltrials.gov. Accessed 3/6, 2025. https://clinicaltrials.gov/study/NCT04750954.

[82] Chauhan, A. et al. Abstract CT194: ETCTN 10388: a first in human phase I trial of triapine and lutetium Lu 177 DOTATATE in well-differentiated somatostatin receptor-positive gastroenteropancreatic neuroendocrine tumors (GEP-NETs). *Cancer Res.* **83**, CT194–CT194 (2023).

[83] Chauhan, A. et al. Pharmacokinetics and RP2D analysis from ETCTN 10388: A phase I trial of triapine and lutetium Lu-177 dotatate in well-differentiated somatostatin receptor–positive gastroenteropancreatic neuroendocrine tumors (GEP-NETs). *J. Clin. Oncol.* **41**, 648 (2023).

[84] Saxton, R. A. & Sabatini, D. M. mTOR signaling in growth, metabolism, and disease. *Cell* **168**, 960–976 (2017). 28283069 10.1016/j.cell.2017.02.004 PMC5394987

[85] Ma, Y., Vassetzky, Y. & Dokudovskaya, S. mTORC1 pathway in DNA damage response. *Biochim Biophys. Acta Mol. Cell Res* **1865**, 1293–1311 (2018). 29936127 10.1016/j.bbamcr.2018.06.011

[86] Yao, J. C. et al. Everolimus for advanced pancreatic neuroendocrine tumors. *N. Engl. J. Med*. **364**, 514–523 (2011). 21306238 10.1056/NEJMoa1009290 PMC4208619

[87] Johnbeck, C. B. et al. 18F-FDG and 18F-FLT-PET imaging for monitoring everolimus effect on tumor-growth in neuroendocrine tumors: studies in human tumor xenografts in mice. *PLoS One* **9**, e91387 (2014). 24626055 10.1371/journal.pone.0091387 PMC3953383

[88] Pool, S. E. et al. mTOR inhibitor RAD001 promotes metastasis in a rat model of pancreatic neuroendocrine cancer. *Cancer Res* **73**, 12–18 (2013). 23149918 10.1158/0008-5472.CAN-11-2089

[89] Aljubran, A. H. et al. Combination of everolimus and lu-177 PRRT in treatment of G1-2 neuroendocrine tumors (NET): Phase 1-2 study. *J. Clin. Oncol.* **37**, 386–386 (2019).30589600

[90] Raymond, E. et al. Sunitinib malate for the treatment of pancreatic neuroendocrine tumors. *N. Engl. J. Med.* **364**, 501–513 (2011). 21306237 10.1056/NEJMoa1003825

[91] Aggarwal, P. et al. Sunitinib in tandem with 177 Lu-DOTATATE therapy in advanced pancreatic neuroendocrine tumor : A new treatment approach. *Clin. Nucl. Med* **49**, e85–e86 (2024). 38109041 10.1097/RLU.0000000000005018

[92] Melincovici, C. S. et al. Vascular endothelial growth factor (VEGF) - key factor in normal and pathological angiogenesis. *Rom. J. Morphol. Embryol.* **59**, 455–467 (2018). 30173249

[93] Wang, H. et al. Response to single low-dose (177)Lu-DOTA-EB-TATE treatment in patients with advanced neuroendocrine neoplasm: A prospective pilot study. *Theranostics* **8**, 3308–3316 (2018). 29930731 10.7150/thno.25919 PMC6010978

[94] Tian, R. et al. Evans blue attachment enhances somatostatin receptor subtype-2 imaging and radiotherapy. *Theranostics* **8**, 735–745 (2018). 29344302 10.7150/thno.23491 PMC5771089

[95] Hänscheid, H., Hartrampf, P. E., Schirbel, A., Buck, A. K. & Lapa, C. Intraindividual comparison of [177Lu]Lu-DOTA-EB-TATE and [177Lu]Lu-DOTA-TOC. *Eur. J. Nucl. Med. Mol. Imaging* **48**, 2566–2572 (2021). 33452632 10.1007/s00259-020-05177-z PMC8241641

[96] Liu, Q. et al. Dose escalation of an Evans blue-modified radiolabeled somatostatin analog (177)Lu-DOTA-EB-TATE in the treatment of metastatic neuroendocrine tumors. *Eur. J. Nucl. Med Mol. Imaging* **47**, 947–957 (2020). 31832728 10.1007/s00259-019-04530-1 PMC7080608

[97] Harris, P. E. & Zhernosekov, K. The evolution of PRRT for the treatment of neuroendocrine tumors; What comes next? *Front Endocrinol. (Lausanne)* **13**, 941832 (2022). 36387893 10.3389/fendo.2022.941832 PMC9659917

[98] Jiang, Y. et al. Safety and efficacy of peptide receptor radionuclide therapy with (177)Lu-DOTA-EB-TATE in patients with metastatic neuroendocrine tumors. *Theranostics* **12**, 6437–6445 (2022). 36185603 10.7150/thno.77219 PMC9516233

[99] Bodei L. A Phase I Study of 177Lu-DOTA-EB-TATE in people with advanced neuroendocrine cancers. Phase 1 study. https://www.mskcc.org/cancer-care/clinical-trials/21-362.

[100] Sundlöv, A. et al. Phase II trial demonstrates the efficacy and safety of individualized, dosimetry-based (177)Lu-DOTATATE treatment of NET patients. *Eur. J. Nucl. Med Mol. Imaging* **49**, 3830–3840 (2022). 35451612 10.1007/s00259-022-05786-w PMC9399027

[101] Sundlöv, A. et al. Phase II trial demonstrates the efficacy and safety of individualized, dosimetry-based 177Lu-DOTATATE treatment of NET patients. *Eur. J. Nucl. Med. Mol. Imaging* **49**, 3830–3840 (2022). 35451612 10.1007/s00259-022-05786-w PMC9399027

[102] Halfdanarson, T.R. et al. COMPOSE: Pivotal Phase III Trial for Well-Differentiated Aggressive Grade 2/3 Gastroenteropancreatic Neuroendocrine Tumors Comparing 177Lu-edotreotide with Best Standard of Care. Endocrine Abstracts. https://www.endocrine-abstracts.org/ea/0089/ea0089t2.

[103] ITM Presents Positive Topline Phase 3 COMPETE Trial Data with n.c.a. 177Lu-edotreotide (ITM-11), a targeted radiopharmaceutical therapy, in patients with grade 1 or 2 gastroenteropancreatic neuroendocrine tumors at the ENETS 2025 Conference. biospace. https://www.biospace.com/press-releases/itm-presents-positive-topline-phase-3-compete-trial-data-with-n-c-a-177lu-edotreotide-itm-11-a-targeted-radiopharmaceutical-therapy-in-patients-with-grade-1-or-2-gastroenteropancreatic-neuroendocrine-tumors-at-the-enets-2025-conference.

[104] Hijioka, S. et al. Current status of medical treatment for gastroenteropancreatic neuroendocrine neoplasms and future perspectives. *Jpn J. Clin. Oncol.* **51**, 1185–1196 (2021). 34038547 10.1093/jjco/hyab076 PMC8326384

[105] La Salvia, A., Espinosa-Olarte, P., Riesco-Martinez, M. D. C., Anton-Pascual, B., Garcia-Carbonero, R. Targeted cancer therapy: What’s new in the field of neuroendocrine neoplasms? *Cancers (Basel)*. 3;13 10.3390/cancers13071701 (2021). 10.3390/cancers13071701 PMC8038369 33916707

[106] Poty, S., Francesconi, L. C., McDevitt, M. R., Morris, M. J. & Lewis, J. S. α-emitters for radiotherapy: From basic radiochemistry to clinical studies-part 2. *J. Nucl. Med* **59**, 1020–1027 (2018). 29496984 10.2967/jnumed.117.204651 PMC6910645

[107] Khazaei Monfared, Y. et al. DNA damage by radiopharmaceuticals and mechanisms of cellular repair. *Pharmaceutics*. 15 10.3390/pharmaceutics15122761 (2023). 10.3390/pharmaceutics15122761 PMC10748326 38140100

[108] Kassis, A. I. & Adelstein, S. J. Radiobiologic principles in radionuclide therapy. *J. Nucl. Med*. **46**, 4s–12s (2005). 15653646

[109] Morgenstern, A. et al. An overview of targeted alpha therapy with (225)actinium and (213)bismuth. *Curr. Radiopharm.* **11**, 200–208 (2018). 29732998 10.2174/1874471011666180502104524 PMC6237921

[110] Sgouros, G. et al. MIRD Pamphlet No. 22 (abridged): Radiobiology and dosimetry of alpha-particle emitters for targeted radionuclide therapy. *J. Nucl. Med* **51**, 311–328 (2010). 20080889 10.2967/jnumed.108.058651 PMC5680544

[111] Makvandi, M. et al. Alpha-emitters and targeted alpha therapy in oncology: From basic science to clinical investigations. *Target Oncol.* **13**, 189–203 (2018). 29423595 10.1007/s11523-018-0550-9

[112] Nayak, T. K. et al. Somatostatin-receptor-targeted alpha-emitting 213Bi is therapeutically more effective than beta(-)-emitting 177Lu in human pancreatic adenocarcinoma cells. *Nucl. Med Biol.* **34**, 185–193 (2007). 17307126 10.1016/j.nucmedbio.2006.11.006

[113] Norenberg, J. P. et al. 213Bi-[DOTA0, Tyr3]octreotide peptide receptor radionuclide therapy of pancreatic tumors in a preclinical animal Model. *Clin. Cancer Res.* **12**, 897–903 (2006). 16467104 10.1158/1078-0432.CCR-05-1264

[114] Kratochwil, C. et al. ²¹³Bi-DOTATOC receptor-targeted alpha-radionuclide therapy induces remission in neuroendocrine tumours refractory to beta radiation: a first-in-human experience. *Eur. J. Nucl. Med Mol. Imaging* **41**, 2106–2119 (2014). 25070685 10.1007/s00259-014-2857-9 PMC4525192

[115] Giesel, F. L. et al. Contrast-enhanced ultrasound monitoring of perfusion changes in hepatic neuroendocrine metastases after systemic versus selective arterial 177Lu/90Y-DOTATOC and 213Bi-DOTATOC radiopeptide therapy. *Exp. Oncol.* **35**, 122–126 (2013). 23828389

[116] Lugat, A. et al. Survival impact of [(225)Ac]Ac-DOTATOC alpha-therapy in a preclinical model of pancreatic neuroendocrine tumor liver micrometastases. *Eur. J. Nucl. Med. Mol. Imaging*. 13;10.1007/s00259-024-06918-0 (2024). 10.1007/s00259-024-06918-0 39269657

[117] Ballal, S., Yadav, M. P., Tripathi, M., Sahoo, R. K., Bal, C. Survival outcomes in metastatic gastroenteropancreatic neuroendocrine tumor patients receiving concomitant (225)Ac-DOTATATE targeted alpha therapy and capecitabine: A real-world scenario management based long-term outcome study. *J. Nucl. Med*. 21;10.2967/jnumed.122.264043 (2022). 10.2967/jnumed.122.264043 35863893

[118] Morris, M. et al. ACTION-1 phase Ib/3 trial of RYZ101 in somatostatin receptor subtype 2–expressing (SSTR2+) gastroenteropancreatic neuroendocrine tumors (GEP-NET) progressing after 177Lu somatostatin analogue (SSA) therapy: Initial safety analysis. *J. Clin. Oncol.* **41**, 4132–4132 (2023).

[119] Strosberg, J. Phase 1b portion of the ACTION-1 phase 1b/3 trial of RYZ101 in gastroenteropancreatic neuroendocrine tumors (GEP-NET) progressing after 177Lu somatostatin analogue (SSA) therapy: Safety and efficacy findings. https://meetings.asco.org/abstracts-presentations/241453.

[120] Perrone, E. et al. Impressive response to TANDEM peptide receptor radionuclide therapy with (177)Lu/(225)AcDOTA-LM3 somatostatin receptor antagonist in a patient with therapy-refractory, rapidly progressive neuroendocrine neoplasm of the pancreas. *Diagnostics (Basel)*. 26;14 10.3390/diagnostics14090907 (2024). 10.3390/diagnostics14090907 PMC11083426 38732321

[121] Delpassand, E. S. et al. Targeted α-emitter therapy with (212)Pb-DOTAMTATE for the treatment of metastatic SSTR-expressing neuroendocrine tumors: First-in-humans dose-escalation clinical trial. *J. Nucl. Med.* **63**, 1326–1333 (2022). 34992153 10.2967/jnumed.121.263230 PMC9454455

[122] Strosberg, J. R. et al. Safety, tolerability and efficacy of 212Pb-DOTAMTATE as a targeted alpha therapy for subjects with unresectable or metastatic somatostatin receptor-expressing gastroenteropancreatic neuroendocrine tumors (SSTR+ GEP-NETs): A phase 2 study. *J. Clin. Oncol.* **42**, 4020–4020 (2024).

[123] Prasad, V. et al. A Phase I/IIa of [212Pb]VMT-a-NET targeted alpha-particle therapy for advanced SSTR2 positive neuroendocrine tumors. *J. Nucl. Med*. **65**, 242430 (2024).

[124] Sen, I., Malik, D., Thakral, P. & Schultz, M. 212Pb-VMT-Î±-NET targeted alpha therapy in metastatic neuroendocrine tumors: First in human study on safety and efficacy. *J. Nucl. Med*. **65**, 242556 (2024). 10.1097/RLU.0000000000005190 38537249

[125] Hooijman, E. L. et al. Implementing Ac-225 labelled radiopharmaceuticals: practical considerations and (pre-)clinical perspectives. *EJNMMI Radiopharm. Chem.* **9**, 9 (2024). 38319526 10.1186/s41181-024-00239-1 PMC10847084

[126] Poty, S., Francesconi, L. C., McDevitt, M. R., Morris, M. J. & Lewis, J. S. α-emitters for radiotherapy: From basic radiochemistry to clinical studies-Part 1. *J. Nucl. Med* **59**, 878–884 (2018). 29545378 10.2967/jnumed.116.186338 PMC6004557

[127] Tosato, M. et al. Alpha Atlas: Mapping global production of α-emitting radionuclides for targeted alpha therapy. *Nucl. Med. Biol.* **142-143**, 108990 (2025). 39809026 10.1016/j.nucmedbio.2024.108990

[128] Shober, M. Regulating alpha-emitting radioisotopes and specific considerations for actinium-225 containing actinium-227. *Appl. Radiat. Isotopes* **187**, 110337 (2022). 10.1016/j.apradiso.2022.110337 35777201

[129] Miederer, M. et al. Alpha-emitting radionuclides: Current status and future perspectives. *Pharmaceuticals* **17**, 76 (2024). 38256909 10.3390/ph17010076 PMC10821197

[130] Elgqvist, J., Frost, S., Pouget, J. P. & Albertsson, P. The potential and hurdles of targeted alpha therapy - clinical trials and beyond. *Front Oncol.* **3**, 324 (2014). 24459634 10.3389/fonc.2013.00324 PMC3890691

[131] Ginj, M. et al. Radiolabeled somatostatin receptor antagonists are preferable to agonists for in vivo peptide receptor targeting of tumors. *Proc. Natl. Acad. Sci. USA* **103**, 16436–16441 (2006). 17056720 10.1073/pnas.0607761103 PMC1618814

[132] Minczeles, N. S., Hofland, J., de Herder, W. W. & Brabander, T. Strategies towards improving clinical outcomes of peptide receptor radionuclide therapy. *Curr. Oncol. Rep.* **23**, 46 (2021). 33721105 10.1007/s11912-021-01037-7 PMC7960621

[133] Lin, Z. et al. Head-to-head comparison of (68)Ga-NODAGA-JR11 and (68)Ga-DOTATATE PET/CT in patients with metastatic, well-differentiated neuroendocrine tumors: Interim analysis of a prospective bicenter study. *J. Nucl. Med* **64**, 1406–1411 (2023). 37474267 10.2967/jnumed.122.264890

[134] Nicolas, G. P. et al. Sensitivity comparison of (68)Ga-OPS202 and (68)Ga-DOTATOC PET/CT in patients with gastroenteropancreatic neuroendocrine tumors: A prospective phase II imaging study. *J. Nucl. Med*. **59**, 915–921 (2018). 29191855 10.2967/jnumed.117.199760

[135] Baum, R. P., Zhang, J., Schuchardt, C., Müller, D. & Mäcke, H. First-in-humans study of the SSTR antagonist (177)Lu-DOTA-LM3 for peptide receptor radionuclide therapy in patients with metastatic neuroendocrine neoplasms: Dosimetry, safety, and efficacy. *J. Nucl. Med*. **62**, 1571–1581 (2021). 33674401 10.2967/jnumed.120.258889 PMC8612334

[136] Zhu, W. et al. A prospective randomized, double-blind study to evaluate the diagnostic efficacy of (68)Ga-NODAGA-LM3 and (68)Ga-DOTA-LM3 in patients with well-differentiated neuroendocrine tumors: compared with (68)Ga-DOTATATE. *Eur. J. Nucl. Med Mol. Imaging* **49**, 1613–1622 (2022). 34874478 10.1007/s00259-021-05512-y

[137] Viswanathan, R. et al. Head-to-Head Comparison of SSTR Antagonist [(68)Ga]Ga-DATA(5m)-LM4 with SSTR Agonist [(68)Ga]Ga-DOTANOC PET/CT in patients with well differentiated gastroenteropancreatic neuroendocrine tumors: A prospective imaging study. *Pharmaceuticals (Basel)*. 22;17 10.3390/ph17030275 (2024). 10.3390/ph17030275 PMC10974918 38543061

[138] Zhang, J. et al. First-in-human study of an optimized, potential kit-type, SSTR Antagonist ^68^Ga-DATA^5m^-LM4 in patients with metastatic neuroendocrine tumors. *J. Nucl. Med.* **15**, 2510–2522 (2025). 10.7150/thno.94521 PMC11840726 39990220

[139] Bodei, L. et al. PRRT neuroendocrine tumor response monitored using circulating transcript analysis: The NETest. *Eur. J. Nucl. Med Mol. Imaging* **47**, 895–906 (2020). 31838581 10.1007/s00259-019-04601-3 PMC7515632

[140] CORRIGENDUM FOR “The inflammation-based index can predict response and improve patient selection in NETs treated with PRRT: A pilot study”. *J. Clin. Endocrinol. Metab.* **104**:2151-2151. 10.1210/jc.2019-00844 (2019). 10.1210/jc.2019-00844 30985879

[141] Bergsma, H. et al. Persistent hematologic dysfunction after peptide receptor radionuclide therapy with (177)Lu-DOTATATE: Incidence, course, and predicting factors in patients with gastroenteropancreatic neuroendocrine tumors. *J. Nucl. Med*. **59**, 452–458 (2018). 28775205 10.2967/jnumed.117.189712

